# A novel missense variant in *cathepsin C* gene leads to PLS in a Chinese patient: A case report and literature review

**DOI:** 10.1002/mgg3.1686

**Published:** 2021-05-05

**Authors:** Hui Yu, Xun He, Xiangqin Liu, Houbin Zhang, Zhu Shen, Yi Shi, Xiaoqi Liu

**Affiliations:** ^1^ College of Medical Technology Chengdu University of Traditional Chinese Medicine Chengdu China; ^2^ Jiangyou People's Hospital Jiangyou China; ^3^ School of Medicine University of Electronic Science and Technology of China Chengdu China; ^4^ Sichuan Provincial Institute of Dermatology and Venereology Chengdu China; ^5^ Department of Dermatology Sichuan Academy of Medical Sciences & Sichuan Provincial People's Hospital Chengdu China; ^6^ Department of Laboratory Medicine Sichuan Academy of Medical Sciences & Sichuan Provincial People's Hospital Chengdu China; ^7^ Sichuan Provincial Key Laboratory for Human Disease Gene Study, Research Unit for Blindness Prevention of Chinese Academy of Medical Sciences (2019RU026) Sichuan Academy of Medical Sciences & Sichuan Provincial People's Hospital Chengdu China

**Keywords:** *Cathepsin C*, missense variant, Papilon‐Lefevre syndrome

## Abstract

**Background:**

Papilon‐Lefevre syndrome (PLS; OMIM 245000) is a rare autosomal recessive disease characterized by aggressive periodontitis and palmoplantar keratoderma. The prevalence of PLS in the general population is one to four cases per million. Although the etiology and pathogenic mechanisms underlying PLS remain largely unclear, existing evidence shows loss‐of‐function mutations of the *cathepsin C* gene (*CTSC*; OMIM 602365) could cause PLS. Here we found a novel variant of the *CTSC* gene in a Chinese PLS family and predicted the effect of the variant on the physic–chemical characters and tertiary structure of the protein.

**Methods:**

The 1–7 coding exons and exon–intron boundaries of *CTSC* gene of the proband and her family were amplified and sequenced directly, and Chromas was used to read sequencing files. Furthermore, the PolyPhen‐2, PROVEAN, and Mutation Taster were utilized to predict the pathogenicity of the variant. Besides, the physic–chemical and structural characters of the protein were analyzed by ProtParam, ProtScale, and SWISS‐MODEL.

**Results:**

Our study identified a novel homozygous variant c.763T>C (p.Cys255Arg) in exon 6 of the *CTSC* gene, and it was a likely pathogenic variant as predicted by PolyPhen‐2, PROVEAN, and Mutation Taster. Moreover, ProtParam and Protscale revealed the variant increased the isoelectric point and hydrophilicity of the protein, and the SWISS‐MODEL analysis suggested the variant was located in a critical domain for protein activity.

**Conclusion:**

Our study analyzed a Chinese family with PLS and identified a novel missense variant in the *CTSC* gene. Besides, this study retrospectively summarized 113 variants of *CTSC* in the world and highlighted the features of 27 *CTSC* variants in Chinese PLS patients. In addition, this study paid much particular attention to the relationship between *CTSC* variants and different phenotypes.

## Graphical abstract

Our study identified a novel missense variant in the *cathepsin C* (*CTSC*) gene. Besides, we summarized variants of CTSC in the world and analyzed the features of CTSC in Chinese Papilon‐Lefevre syndrome patients. Furthermore, we paid specific attention to the relationship between CTSC variants and different phenotypes.

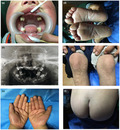

## 1 INTRODUCTION

Papilon‐Lefevre syndrome (PLS; OMIM 245000), categorized into type IV ectodermal dysplasia of palmoplantar, is an uncommon autosomal recessive inheritance and first reported in 1924 by Papillon and Lefevre (Papillon & Lefevre, 1924). The prevalence of PLS in the general population is about one to four cases per million and parental consanguinity accounts for more than 50% PLS cases (Pap et al., 2020). The dominant clinical symptoms of PLS are symmetrical and erythematous palmoplantar hyperkeratosis, which often worsen in autumn and winter and result in difficulties in walking and physical activities. What's more, hyperkeratosis can gradually progress other parts of the body, such as the elbows, knees, ankles, and knuckles (Romero‐Quintana et al., 2013). Besides, PLS patients often have rapid and severe early‐onset periodontitis and gingivitis, which lead to premature loss of primary and permanent teeth and seriously influence chew function and facial contour (Wei et al., 2020). In addition to these basic features, patients with PLS also can present with mild mental retardation, intracranial calcifications, nail dystrophy, etc. About 20%–25% of PLS patients increased susceptibility to microbial infections, such as skin abscess, kidney abscess, or liver abscess (Wu et al., 2016, 2019).

Although antibiotic therapy and surgical treatment can effectively reduce clinical symptoms and delay tooth loss, there is no successful and definitive treatment protocol in managing patients with PLS. It is necessary to explore etiological research to establish correct and precise diagnosis and treatment management. Existing evidence shows the etiology and pathogenic mechanisms underlying PLS are very complex, and the loss‐of‐function mutations of *cathepsin C* gene (*CTSC*; OMIM 602365) are the main genetic contributor to PLS. *CTSC* belongs to the member of lysosomal cysteine protease of the papain superfamily of cysteine proteases and has a great impact on mediating cytotoxicity, destructing bacteria, and regulating the activation of a serine protease (Meenu et al., 2020). To date, 113 *CTSC* variants have been reported in ethnically various populations. Among them, the vast majority of *CTSC* variants are closely related to PLS.

According to the clinical manifestations and laboratory examinations of the proband, we suspected that the proband was a PLS patient, so we carried out *CTSC* gene variant screening by Sanger sequencing. In fact, in addition to using Sanger sequencing to screen *CTSC* gene variants, whole‐exome sequencing also can be used to identify pathogenic variants efficiently. and it can screen for molecular confirmation of all related genes quickly (Alkhiary et al., 2016). In this study, we found a novel homozygous variant in exon 6 of the *CTSC* gene. The effects of the variant on physical and chemical properties and tertiary structure were predicted by bioinformatics tools. What's more, we retrospectively summarized 113 variants of *CTSC* in the world and mainly analyzed the features of 27 *CTSC* variants in Chinese PLS patients. Besides, specific attention had been paid to the relationship between *CTSC* variants and different phenotypes. This study could help enlarge the scope of *CTSC* gene variants associated with PLS and expanding the knowledge on complex genotype‐to‐phenotype relationships of *CTSC* pathogenicity.

## 2 MATERIALS AND METHODS

### 2.1 Editorial policies and ethical considerations

The Institution Review Board and the Ethics Committee Sichuan Provincial People's Hospital formally approved this study. Written informed consent of the subjects detailed in this study in accordance with the Declaration of Helsinki was obtained from adult participants and the patient guardians.

### 2.2 Subjects

The proband was a PLS patient from Yi ethnic group in China, female, 5 years. A four‐generation pedigree was recruited in our study. One patient and eight members of the family were examined for medicine and genetic research. The family history revealed that her family was normal and her parents had consanguineous marriage. The PLS diagnosis was mainly based on clinical manifestations, laboratory tests, and other features.

### 2.3 Mutation screening

We extracted and quantified genomic DNAs by standard technology using DNA extraction Kits (Qiagen, Hilden, Germany) from peripheral blood leukocytes of the proband and her available family members after obtaining informed consent. The *CTSC* (GenBank reference sequence: NM_001814.6) primer sequences of exon 1 to 7 were shown in Table S1. The coding exon sequences and nearby exon–intron borders of the *CTSC* gene were amplified by PCR technology (Thermal cycler, USA), and the reactions were conducted in a 20 µl mixture containing 6 µl of ddH_2_O, 10 µl of 2×Taq Master Mix, 1 µl of each primer, and 2 µl of genomic DNA template. The PCR reaction conditions were as follows: initial denaturation at 94℃ for 2 min; then 35 cycles of 94℃ for 30 s, 58℃ for 30 s, 72℃ for 30 s, and a final elongation step at 72℃ for 5 min. Finally, the purified PCR products were directly sequenced using ABI PRISM 3730xL DNA Analyzers (Applied Biosystems, USA).

### 2.4 Bioinformatic analysis

PolyPhen‐2 (http://genetics.bwh.harvard.edu/pph2/) (Adzhubei et al., 2010), PROVEAN (http://provean.jcvi.org/index.php) (Choi et al., 2012), and Mutation Taster (http://mutationtaster.org/) (Schwarz et al., 2014) were used to predict the functional effects and pathogenicity of the variant. ProtParam (https://web.expasy.org/protparam/) and ProtScale (https://web.expasy.org/protscale/) were utilized to determined the physical and chemical properties of the protein. SWISS‐MODEL and PyMOL were applied to construct and analyze models of protein structure.

## 3 RESULTS

### 3.1 Clinical findings

**FIGURE 1:**
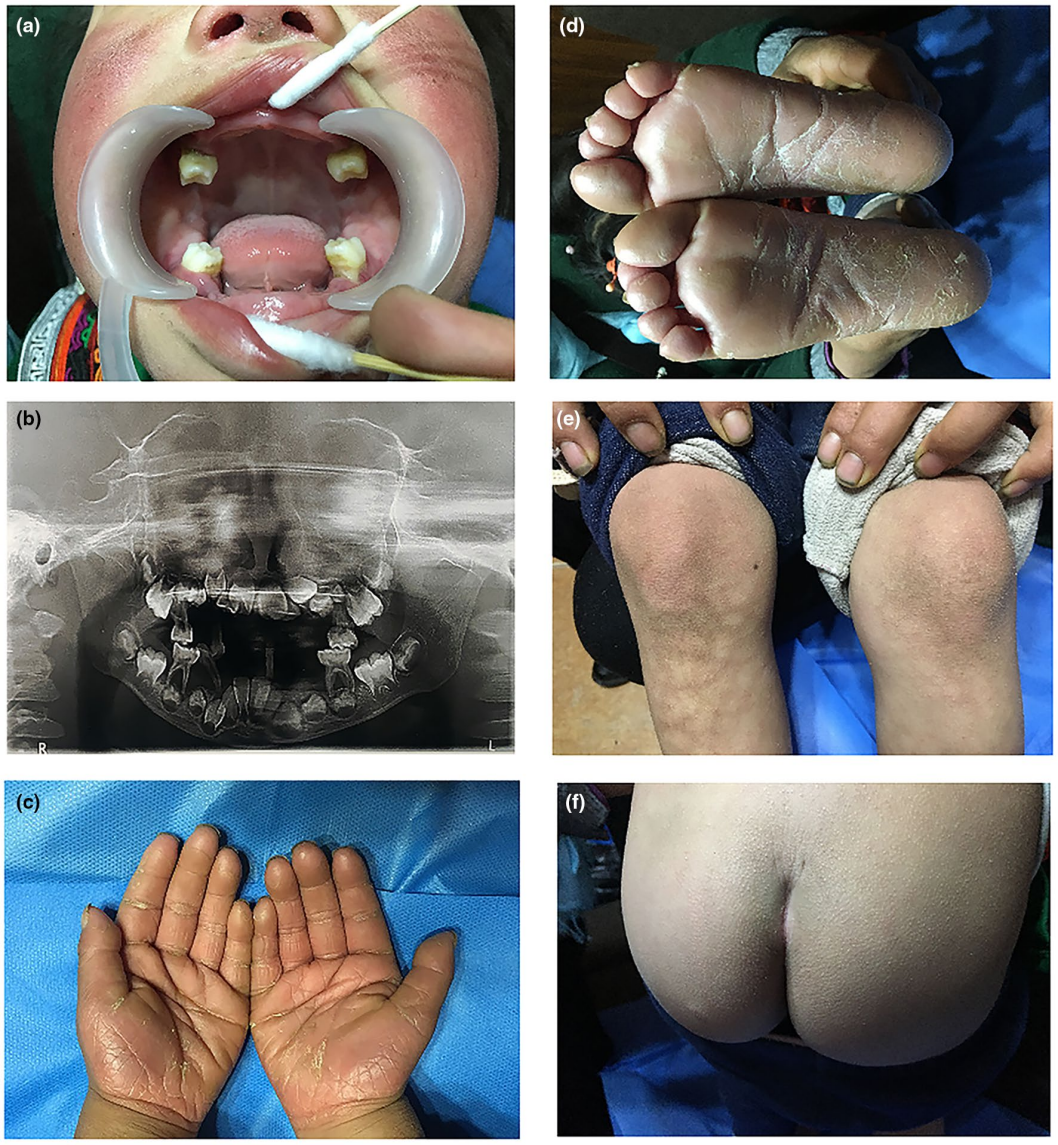
Clinical features of the proband. The patient presented with nearly complete loss of her teeth, gingivitis, and abnormal mucosa (a). Panoramic radiographic examination showed that the alveolar occurred extensive bone absorption (b). The palm (c) and sole (d) revealed hyperkeratosis. The knees (e) and buttocks (f) showed mild hyperkeratosis

Examination of the oral cavity indicated loss of majority of teeth, loose the remaining teeth, red and swollen gums, abnormal mucosa, and no fester (Figure 1a). Panoramic radiographic examination revealed severe resorption of the bone and generalized alveolar bone loss (Figure 1b). Physical examination showed the skin of palms and soles of both sides of the patient were hyperkeratosis, accompanied by skin thickening and chapping (Figure 1c,d). The knees and buttocks also showed slight keratosis (Figure 1e,f), not involving the dorsal side of hands and feet, ankles, and elbows. There were no signs of aggressive periodontitis and hyperkeratosis of palms and soles in her parents and brothers.

### 3.2 Sequencing analysis

**FIGURE 2:**
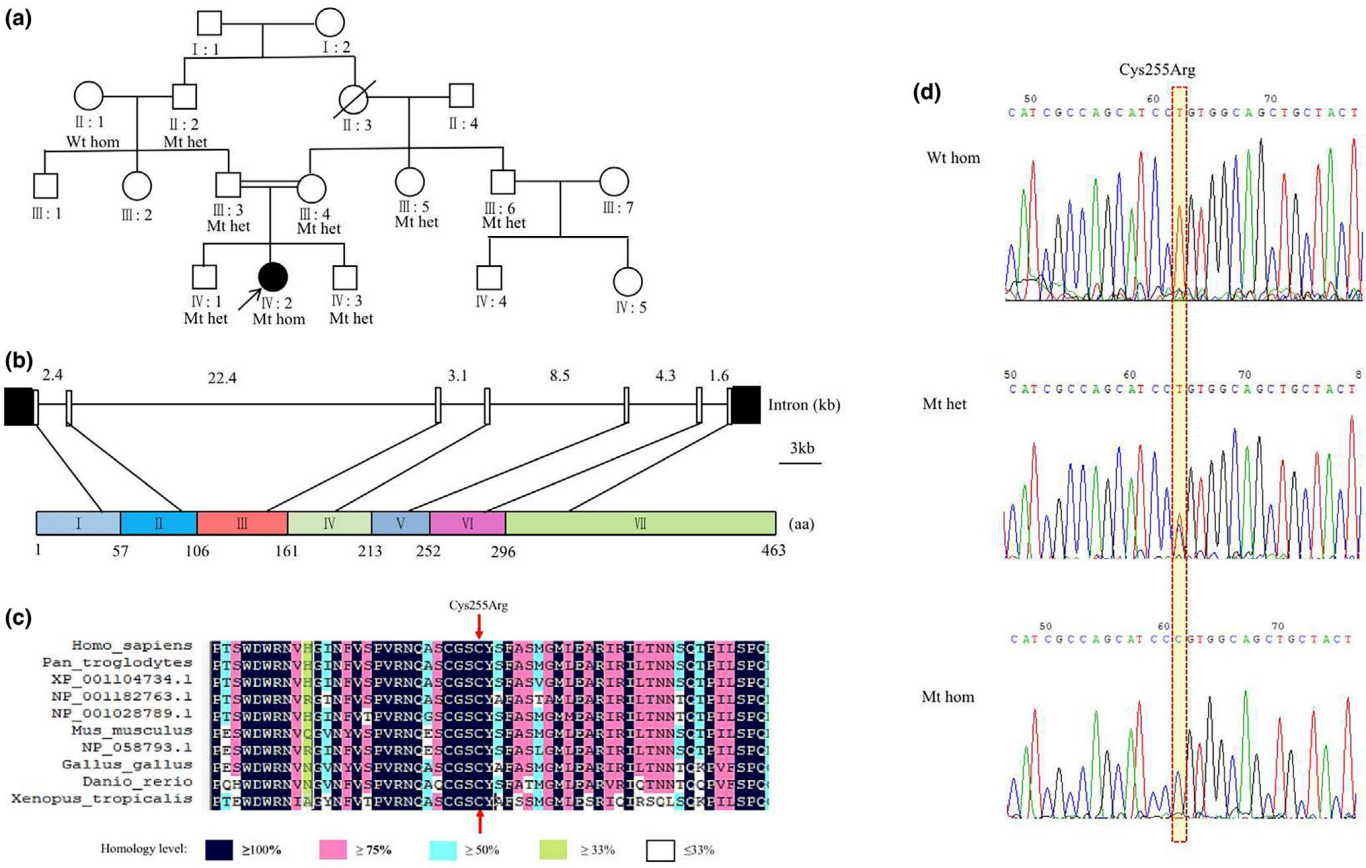
Pedigree diagram of the Chinese family with PLS (a). Sequence analysis of exon 6 of the *cathepsin C* gene. Wild‐type unaffected individuals showed normal sequence. Carriers showed a heterozygous mutation. The affected individual showed a homozygous variant with a T>C change at nucleotide 763 (d). Het, heterozygous; WT, wild‐type; Wt hom, wild‐type homozygous; Mt het: heterozygous Mutation; Mt homozygous: homozygous mutation. Multiple‐sequence alignment of CTSC proteins from different species, and the 255th amino acid residue is located in exon 6 of the heavy chain and is highly conserved among species (b) and (c)

The 1–7 coding exons and exon–intron boundaries of the *CTSC* gene of the proband and her family were sequenced and analyzed. The proband showed a novel homozygous missense variant at base position 763 in exon 6 of the *CTSC* gene, and base T was replaced by C (c.763T>C). The TGT codon encoding Cysteine was changed to CGT codon encoding Arginine (p.Cys255Arg). The proband's parents, two brothers, and grandfathers carried a heterozygous variant of *CTSC* gene c.763T>C (Figure 2a,d).

### 3.3 Bioinformatic analysis

Multiple sequence alignment showed that the 255th amino acid in exon 6 was highly conserved among species (Figure 2b,c). The variant was predicted to be “probably damaging,” “deleterious,” and “disease causing” by PolyPhen‐2, PROVEAN, and Mutation Taster respectively.

**FIGURE 3:**
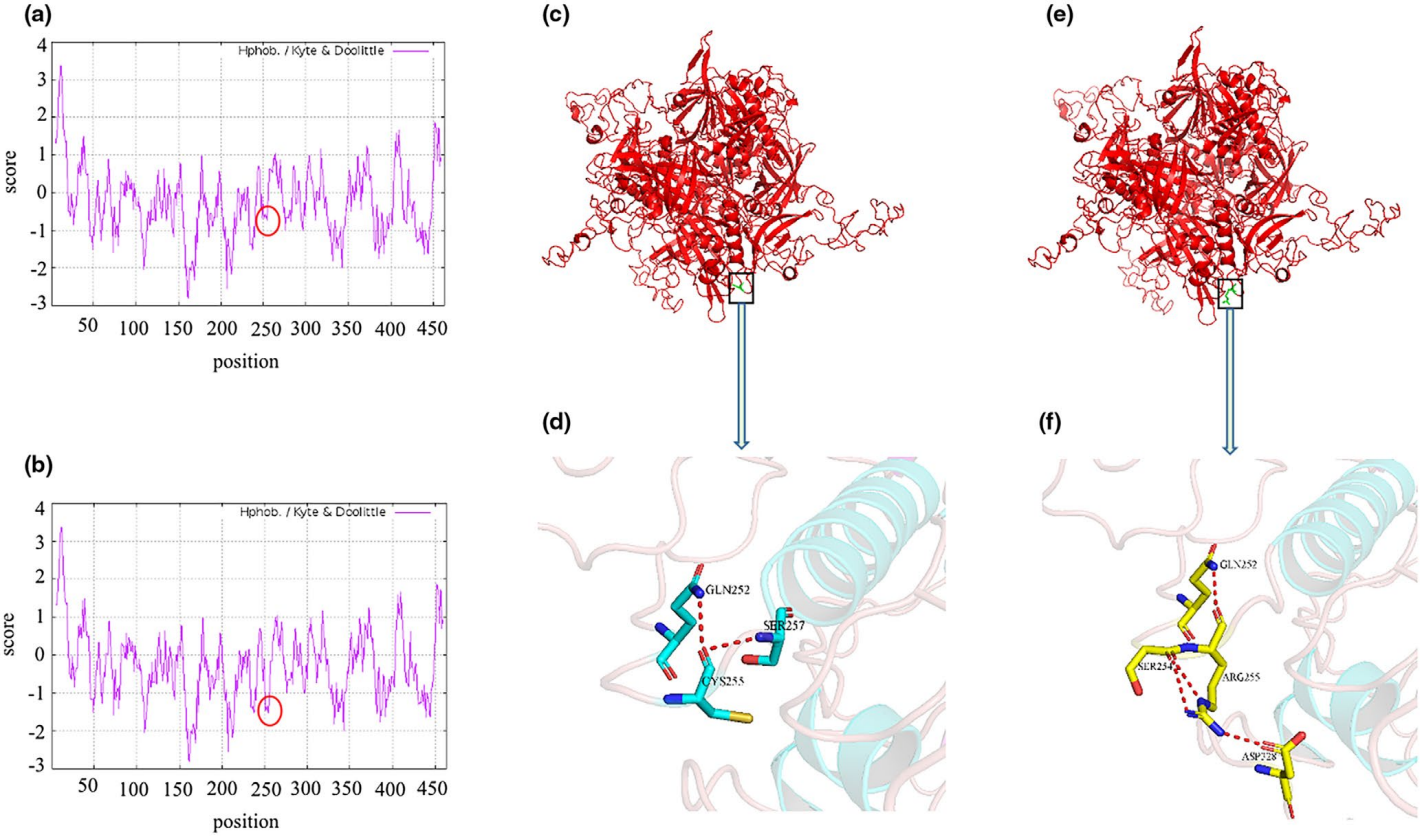
Protscale analysis of wild‐type and mutant CTSC protein. (a) Wild‐type, (b) Mutant. The score of the Cys255Arg site significantly decreased. The tertiary structure of wild‐type CTSC protein (c) and its interaction residues (d). The tertiary structure of mutant‐type CTSC protein (e) and its interaction residues (f). The 255th amino acid is located in a special position, which is near the helix. The 255th residue of wild‐type can interact with residues 252 and 257 in the sequence space respectively. The 255th residue of mutant type can interact with 252, 254, and 328 in the sequence space

ProtParam indicated that variant protein showed a modest increase in the theoretical pI and showed a modest decrease in the instability index and grand average of hydropathicity (GRAVY) (Table 1). ProtScale revealed the score of the variation site decreased significantly, meaning the hydrophilicity increased significantly (Figure 3a,b).

**TABLE 1:** ProtParam analysis of wild‐type and mutant CTSC protein

	Molecular weight (Daltons)	Theoretical pI	Instability index^a^	Aliphatic index	Grand average of hydropathicity (GRAVY)^b^
Wild‐type	51,853.82	6.53	36.05	74.34	−0.257
Mutant	51,906.87	6.70	35.57	74.34	−0.273

^a^ Instability index: the instability index is >40, it predicts the protein is unstable; the instability index is <40, it predicts the protein is stable.

^b^ The negative value of GRAVY stands for hydrophilic protein and lower negative value represents higher hydrophilicity.

The crystal structure of the wild type CTSC protein (PDB ID: 3PDF), obtained from the RCSB‐PDB database (http://www.rcsb.org/pdb), was used as a template to constructed variant protein. SWISS‐MODEL and PyMOL were used to analyze the effect of the variant on the tertiary structure of CTSC protein. It can be seen from Figure 3c–f that the variant was located in a special position, which may break a central helix.

## 4 DISCUSSION

PLS is a kind of early‐onset, severe, and aggressive disease, which seriously affects the patients’ quality of life as well as physical and mental health. The deficiency of the *CTSC* gene is considered to be closely related to PLS. The *CTSC* is highly expressed in lymphocytes, natural killer cells, and other immune cells, the variants of the gene may impair the immune response, which could possibly explain susceptibility to microbial infections in PLS patients. Moreover, the *CTSC* plays an important role in epithelial proliferation and degradation, variants of the *CTSC* gene perhaps cause epithelial cells to be in a hyperproliferative state, such as observed on the palms and soles of PLS patients (Kurban et al. ,^2009^, ^2010^). Besides, *CTSC* is also involved in abnormal differentiation of epithelial cells, variants of the *CTSC* gene probably affect adhesion between gingiva and tooth surface, weakening the mechanical barrier against pathogenic organisms and leading to the destruction of periodontal tissue and loss of teeth (Wu et al., 2019). In a word, *CTSC* variants could affect immune response and proliferation and differentiation of epithelial cells in PLS patients.

**FIGURE 4:**
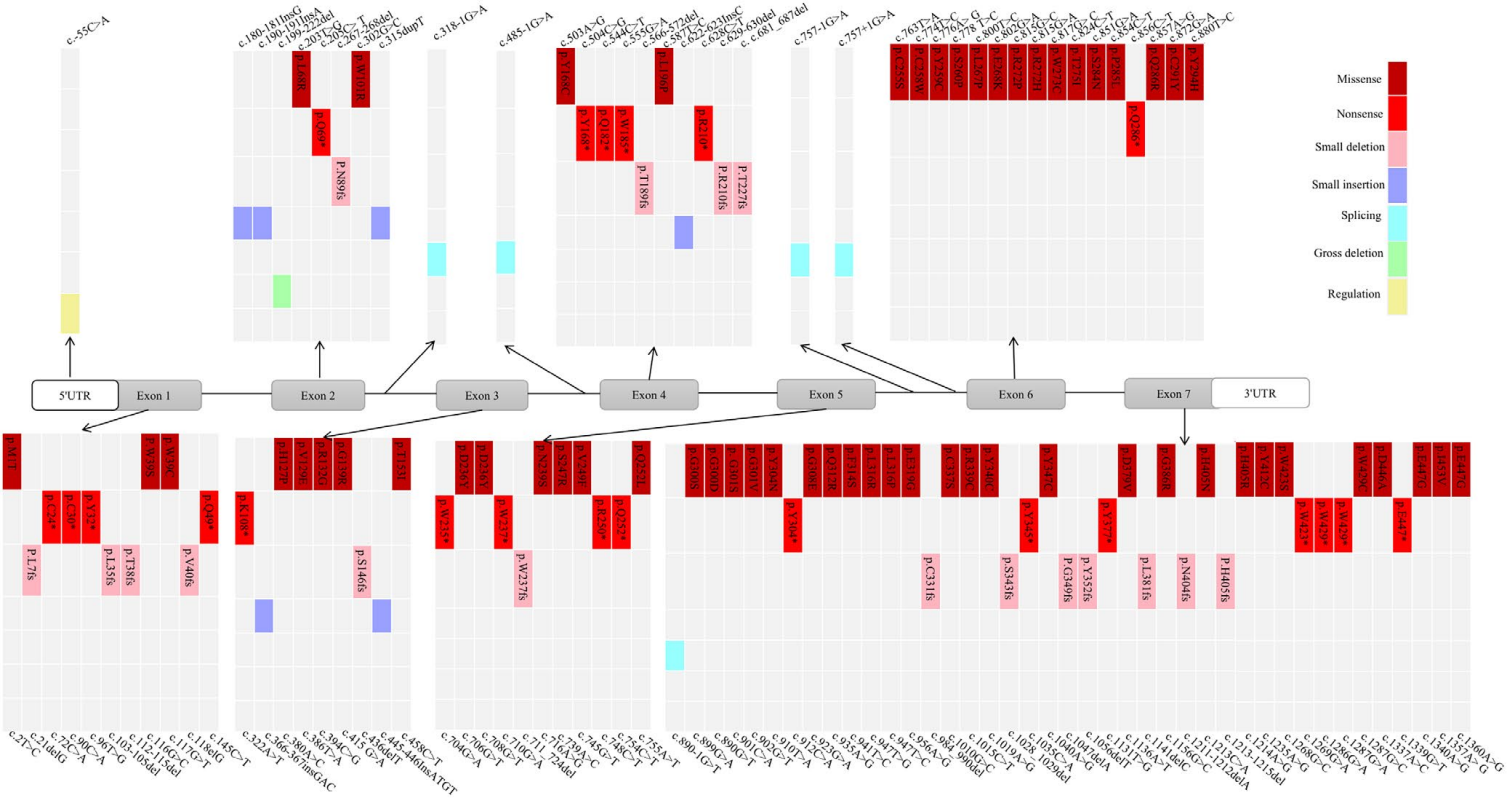
The distribution of *CTSC* variants reported around the world. Another three gross deletions, Chr11:g.88032292‐88142997del, Gene deletion exons 3–7 and Chr11:g.88016961‐88165581del are not shown

Up to now, 113 different *CTSC* gene variants have been reported in different ethnic backgrounds (as shown in Table S2). The distribution of variants was shown in Figure 4. The majority of PLS patients carried *CTSC* gene variants in either homozygous or compound heterozygous forms. Individuals harboring only one heterozygous variant of *CTSC* usually did not show any PLS or related disease phenotypes. There was no evidence for a link between the different types of variants and the severity and site of hyperkeratosis by analyzing the clinical symptoms of PLS. Surprisingly, PLS patients with nonsense or del‐ins variations had generally decreased CTSC enzyme activity (Hart, Zhang, et al., 2000; Toomes et al., 1999; Wu et al., 2019). Among the 113 *CTSC* gene variants, c.815G>C, c.901C>A, c.628C>T, and c.748C>T were high‐frequency variants and widely distributed around the world. Besides, more than 90% of these variants, mainly located in exon 5–7 of the CTSC protein, were missense variants, nonsense variants, or frameshift variants. These variants may damage the formation of the papain‐like domain, or prevent CTSC protein from being transported to the appropriate organelles, or prevent the protein kinase from being effectively activated (Turk et al., 2001).

In our study, the Chinese patient who carried a novel missense variant in exon 6 of *CTSC* (c.763T>C). To our best knowledge, the variant has not been reported so far according to the public database of the 1000 Genomes Project (https://www.internationalgenome.org/1000‐genomes‐browsers/). This variant was highly conserved across different species and suspected to be disease causing with high probability by PolyPhen‐2, PROVEAN, and Mutation Taster. Besides, this missense variant was analyzed by Protparam and ProtScale to predict its effect on physical and chemical properties, the results illustrated that this variant changed the stability and hydrophilicity of the protein, thus probably changing the activity and specificity of CTSC enzyme. Moreover, the predicted spatial structure of the CTSC protein showed the variant residue was located in a special domain. The variant may hinder the formation of the correct specific structure and disturb the local structure. What's more, the variant may affect the internal interactions of the protein or disturb interactions with other molecules, thereby decreasing or abolishing the activity of the CTSC enzyme. In conclusion, this novel variant may reduce the activity of CTSC enzyme leading to clinical symptoms of PLS through several mechanisms such as changing the physical and chemical properties or changing the three‐dimensional structure of the protein.

In addition to the novel variant in this study, 27 different variants of *CTSC* gene were found in Chinese patients with PLS, including 15 missense variants, 5 nonsense variants, 4 frameshift variants, 2 gross deletion variants, and 1 splicing variant. Interestingly, two gross deletion variants were reported in China, wherea a total of four gross deletion variants was reported around the world. More than 70% of the variants were clustered in exons 5, 6, and 7. And most Chinese patients were in a compound heterozygous state. The variation types and distribution of *CTSC* gene in Chinese patients were similar to those reported in the world. Among the Chinese patients with *CTSC* gene variants, one patient showed physical retardation (Wu et al., 2019). It was worth noting that one patient manifested skin abnormalities at 1 week after she was born, and this finding was earlier than most previous reports (Wu et al., 2016). In addition, Wang et al. first reported that serum levels of IgE were significantly increased in a PLS patient and the serum IgE subsequently decreased in tune with the recovery of periodontitis (Wang et al., 2015; Wen et al., 2012).

Through extensive literature analysis, we found that pathogenic variant of *CTSC* gene can lead to three closely related diseases: PLS, Haim‐Munk syndrome (HMS; OMIM 245010), and Aggressive periodontitis (AP1; OMIM 170650). Haim‐Munk syndromeis, an extremely rare autosomal recessive disorder of keratinization associated with aggressive periodontit, is palmoplantar hyperkeratosis, arachnodactyly, onychomycosis, acral osteolysis, atrophic nail changes, and special radiographic deformity of the fingers (Sulak et al., 2016). Aggressive periodontitis is characterized by severe periodontitis but lack of palmoplantar keratosis (Molitor et al., 2019). They have overlapping clinical characteristics, including severe early‐onset periodontitis. Moreover, many studies have reported that PLS, HMS, and AP1 belong to phenotypic variants of the same rare disease caused by allelic variants of *CTSC* gene (Hart, Hart, et al., 2000; Rai et al., 2010). According to our current knowledge, only 5 *CTSC* variants (c.145C>T, c.587T>C, c.748C>T, c.857A>G, and c.1337A>C) were reported in HMS patients, and all of them were also reported in PLS patients. In order to further clarify the relationship between genotypes and different phenotypes, Pap ÉM et al. researched two patients with different phenotypes (PLS and HMS) but harboring the same nonsense *CTSC* variant by using whole‐exome sequencing (WES), the results found two potentially relevant putative phenotype‐modifying variants in HMS patient: a missense variant (rs34608771) of the *SH2D4A* gene and a missense variant (rs55695858) of the *OBP2A* gene, and they affected the intracellular signal transduction of the cystatin F, a known inhibitor of CTSC, and the function of CTSC through GLT6D1, respectively (Pap et al., 2020). However, haplotype analysis of 24 polymorphic markers in *CTSC* gene showed that PLS and HMS patients with the same homozygous nonsense *CTSC* gene variant carried the same haplotype, this result indicated that putative genetic‐modifying factors in the *CTSC* gene were nonexistent or other lifestyle or environmental factors also played important roles in different disease phenotypes observed among patients (Sulak et al., 2016). To understand the relationship between genotypes and phenotypes, it is not only necessary to have a thorough understanding of the mechanisms underlying identified molecular regulatory hubs, but also to understand their effects in one environment or across different environments.

## 5 CONCLUSION

In this study, we sequenced coding exons and exon–intron boundaries of the *CTSC* gene in a Chinese family with classical PLS and found a novel homozygous likely pathogenic variant in exon 6 of *CTSC* (c.763T>C). In addition, we retrospectively summarized published *CTSC* variants and analyzed the features of *CTSC* variants in Chinese PLS patients. And we paid much specific attention to the relationship between *CTSC* variants and different phenotypes. Our study could be helpful in understanding the genetic background of PLS patients and expanding the knowledge on the complexity of genotype‐to‐phenotype relationships of *CTSC* pathogenicity. Moreover, this study can provide important support for diagnosis and genetic counseling for PLS patients and promote individualized clinical treatment.

## CONFLICT OF INTEREST

The authors all report that they have no conflicts of interest in this study.

## AUTHOR CONTRIBUTIONS

Hui Yu, Xun He, and Xiangqin Liu contributed to the acquisition of literature, writing, and revising the manuscript; Houbin Zhang and Yi Shi carried out all experiments and did data analysis; Xiaoqi Liu and Zhu Shen conceived the research and wrote the final manuscript. All of the authors have read and approved the final manuscript.

## Supporting information

Table S1

Table S2

## Data Availability

The data that support the findings of this study are available from the corresponding author upon reasonable request.
