# Supplementary material for: A novel missense variant in cathepsin C gene leads to PLS in a Chinese patient: A case report and literature review
Source: Mol Genet Genomic Med. 2021 May 5;9(7):e1686. doi: 10.1002/mgg3.1686 (PMC8372118; doi:10.1002/mgg3.1686)
Supplement: Supplementary file 1 — Table S1 [file MGG3-9-e1686-s001.docx]

Supplementary Table S1: The primer sequences of CTSC* gene

| Primer name | Primer sequence |
| --- | --- |
| Exon 1 | F：AAATCTGTCCCTGGCCTCTT |
|  | R：AACCAAACGATTGCAGTTCC |
| Exon 2 | F：GACTGTGCTCAAACTGGGTAG |
|  | R：CTACTAATCAGAAGAGGTTTCAG |
| Exon 3 | F：GGGGCACATTTACTGTGAATG |
|  | R：CGTATGTCTCATTTGTAGCAAC |
| Exon 4 | F：GTACCACTTTCCACTTAGGCA |
|  | R：GGAGGATGGTATTCAGCATTC |
| Exon 5 | F：CCTAGCTAGTCTGGTAGCTG |
|  | R：GTATCCCCGAAATCCATCACA |
| Exon 6 | F：CTCTGTGAGGCTTCAGATGTC |
|  | R：CAACAGCCAGCTGCACACAG |
| Exon 7 | F-1：CGGCTTCCTGGTAATTCTTC |
|  | R-1：GTAGTGGAGGAAGTCATCATATAC |
|  | F-2：CAATGAAGCCCTGATCAAGC |
|  | R-2：CTTCTGAGATTGCTGCTGAAAG |

^[[1]](#footnote-0)^F: Forward primer; R: Reversed primer

*: GenBank reference sequence: [NM_001814.6](https://www.ncbi.nlm.nih.gov/nuccore/NM_001814.6)

1. [↑](#footnote-ref-0)
