# Supplementary material for: A novel missense variant in cathepsin C gene leads to PLS in a Chinese patient: A case report and literature review
Source: Mol Genet Genomic Med. 2021 May 5;9(7):e1686. doi: 10.1002/mgg3.1686 (PMC8372118; doi:10.1002/mgg3.1686)
Supplement: Supplementary file 2 — Table S2 [file MGG3-9-e1686-s002.docx]

| Supplementary Table S2: A summary of the reported variants of CTSC gene around the world | | | | | | | | | | | | | | |
| --- | --- | --- | --- | --- | --- | --- | --- | --- | --- | --- | --- | --- | --- | --- |
| Variant | Location | | Amino acid change | Referrence | Ethnicity | Consanguineous | Year |  |  |  |  |  |  |  |
| **Variant type: Missense Variant** | | | |  | | | | |  |  |  |  |  |  |
| c.2T>C | exon 1 | | p.Met1Thr | (Ochiai et al., 2009) | Japanese | Non | 2009 |  |  |  |  |  |  |  |
| c.116G>C | exon 1 | | P.Trp39Ser | (Nakano et al., 2001) | Puerto Rican | Non | 2001 |  |  |  |  |  |  |  |
| c.117G>T | exon 1 | | p.Trp39Cys | (Tekin, Yucelten, Beleggia, Sarig, & Sprecher, 2016) | Turkish | Consanguineous | 2016 |  |  |  |  |  |  |  |
| c.203T>G | exon 2 | | p.Leu68Arg | (Romero-Quintana et al., 2013) | Mexican | Non | 2013 |  |  |  |  |  |  |  |
| c.302G>C | exon 2 | | p.Trp101Ser | (Molitor et al., 2019) | Turkish | Consanguineous | 2019 |  |  |  |  |  |  |  |
| c.380A>C | exon 3 | | p.His127Pro | (Lefevre et al., 2001) | French | Non | 2001 |  |  |  |  |  |  |  |
| c.386T>A | exon 3 | | p.Val129Glu | (Hewitt et al., 2004) | Not | Non | 2004 |  |  |  |  |  |  |  |
| c.394C>G＃ | exon 3 | | p.Arg132Gly | (Wang et al., 2015) | Chinese | Non | 2015 |  |  |  |  |  |  |  |
| c.415G>A＃ | exon 3 | | p.Gly139Arg | (Zhang et al., 2002) | American | Non | 2002 |  |  |  |  |  |  |  |
| c.458C>T | exon 3 | | p.Thr153Ile | (P. S. Hart et al., 2000) | Scottish | Non | 2000 |  |  |  |  |  |  |  |
| c.503A>G | exon 4 | | p.Tyr168Cys | (Sorensen et al., 2014) | European | Non | 2014 |  |  |  |  |  |  |  |
| c.587T>C | exon 4 | | p.Leu196Pro | (Cury et al., 2002) | Brazilian | Consanguineous | 2002 |  |  |  |  |  |  |  |
| c.706G>T | exon 5 | | p.Asp236Tyr | (Allende et al., 2001) | Not | Non | 2001 |  |  |  |  |  |  |  |
| c.716A>G＃ | exon 5 | | p.Asn239Ser | (Y. Wu, Zhao, Xu, & Wu, 2019) | Not | Non | 2019 |  |  |  |  |  |  |  |
| c.739A>C＃ | exon 5 | | p.Ser247Arg | (Wen, Wang, & Duan, 2012) | Chinese | Non | 2012 |  |  |  |  |  |  |  |
| c.745G>T | exon 5 | | p.Val249Phe | (Toomes et al., 1999) | Not | Non | 1999 |  |  |  |  |  |  |  |
| c.755A>T | exon 5 | | p.Gln252Leu | (Toomes et al., 1999) | Not | Non | 1999 |  |  |  |  |  |  |  |
| c.763T>A＃ | exon 6 | | p.Cys255Ser | (X. Li, Zhang, Zhang, & Chen, 2008) | Chinese | Non | 2008 |  |  |  |  |  |  |  |
| c.774T>C＃ | exon 6 | | p.Cys258Trp | (Yang, Bai, Liu, Cao, & Ge, 2006) | Chinese | Non | 2006 |  |  |  |  |  |  |  |
| c.776A>G | exon 6 | | p.Tyr259Cys | (Meenu et al., 2020) | Indian | Consanguineous | 2020 |  |  |  |  |  |  |  |
| c.778 T>C＃ | exon 6 | | p.Ser260Pro | (Yang et al., 2006) | Chinese | Non | 2006 |  |  |  |  |  |  |  |
| c.800T>C＃ | exon 6 | | p.Leu267Pro | (Hu, Zou, & Ye, 2019) | Chinese | Non | 2019 |  |  |  |  |  |  |  |
| c.802G>A | exon 6 | | p.Glu268Lys | (Hayashi, Nakano, Sawamura, & Suzuki, 2020) | Japanese | Non | 2020 |  |  |  |  |  |  |  |
| c.815G>C | exon 6 | | p.Arg272Pro | (Toomes et al., 1999) | Not | Non | 1999 |  |  |  |  |  |  |  |
| c.815G>A | exon 6 | | p.Arg272His | (Hewitt et al., 2004) | Not | Non | 2004 |  |  |  |  |  |  |  |
| c.817G>C | exon 6 | | p.Trp273Cys | (Hattab & Amin, 2005) | Jordanian | Consanguineous | 2005 |  |  |  |  |  |  |  |
| c.824C>T＃ | exon 6 | | p.Thr275Ile | (Z. Li et al., 2014) | Chinese | Non | 2014 |  |  |  |  |  |  |  |
| c.851G>A＃ | exon 6 | | p.Ser284Asn | (Yang et al., 2007) | Chinese | Non | 2007 |  |  |  |  |  |  |  |
| c.854C>T | exon 6 | | p.Pro285Leu | (Noack et al., 2008) | Moroccan | Consanguineous | 2008 |  |  |  |  |  |  |  |
| c.857A>G | exon 6 | | p.Gln286Arg | (P. S. Hart et al., 2000) | Indian | Non | 2000 |  |  |  |  |  |  |  |
| c.872G>A | exon 6 | | p.Cys291Tyr | (Allende et al., 2001) | Not | Non | 2001 |  |  |  |  |  |  |  |
| c.880T>C | exon 6 | | p.Tyr294His | (Allende, Moreno, & de Unamuno, 2003) | Spanish | Non | 2003 |  |  |  |  |  |  |  |
| c.898G>A | exon 7 | | p.Gly300Ser | (P. S. Hart et al., 2000) | Vietnamese | Non | 2000 |  |  |  |  |  |  |  |
| c.899G>A | exon 7 | | p.Gly300Asp | (Zhang et al., 2001) | Arabian | Non | 2001 |  |  |  |  |  |  |  |
| c.901G>A＃ | exon 7 | | p.Gly301Ser | (Toomes et al., 1999) | Iranian | Non | 1999 |  |  |  |  |  |  |  |
| c.902G>T | exon 7 | | p.Gly301Val | (P. S. Hart et al., 2000) | Iranian | Non | 2000 |  |  |  |  |  |  |  |
| c.910T>A | exon 7 | | p.Tyr304Asn | (P. S. Hart et al., 2000) | Panamanian | Non | 2000 |  |  |  |  |  |  |  |
| c.923G>A | exon 7 | | p.Gly308Glu | (Castori et al., 2009) | Italian | Non | 2009 |  |  |  |  |  |  |  |
| c.935A>G | exon 7 | | p.Gln312Arg | (Hewitt et al., 2004) | Not | Non | 2004 |  |  |  |  |  |  |  |
| c.941T>C＃ | exon 7 | | p.Phe314Ser | (X. Li et al., 2008) | Chinese | Non | 2008 |  |  |  |  |  |  |  |
| c.947T>C | exon 7 | | p.Leu316Pro | (Noack et al., 2004) |  | Non | 2004 |  |  |  |  |  |  |  |
| c.947T>G | exon 7 | | p.Leu316Arg | (Noack et al., 2004) | German | Non | 2004 |  |  |  |  |  |  |  |
| c.956A>G | exon 7 | | p.Glu319Gly | (P. S. Hart et al., 2000) | Iranian | Non | 2000 |  |  |  |  |  |  |  |
| c.1010G>C | exon 7 | | p.Cys337Ser | (Machado et al., 2019) | Not | Consanguineous | 2019 |  |  |  |  |  |  |  |
| c.1015C>T＃ | exon 7 | | p.Arg339Cys | (Toomes et al., 1999) | Not | Non | 1999 |  |  |  |  |  |  |  |
| c.1019A>G | exon 7 | | p.Tyr340Cys | (P. S. Hart et al., 2000) | Turkish | Consanguineous | 2000 |  |  |  |  |  |  |  |
| c.1040A>G＃ | exon 7 | | p.Tyr347Cys | (Toomes et al., 1999) | Not | Non | 1999 |  |  |  |  |  |  |  |
| c.1136A>T＃ | exon 7 | | p.Asp379Val |  | Chinese | Non | 2019 |  |  |  |  |  |  |  |
| c.1156G>C | exon 7 | | p.Gly386Arg | (Jouary et al., 2008) | Caucasian | Non | 2008 |  |  |  |  |  |  |  |
| c.1213C>A | exon 7 | | p.His405Asn | (de Haar et al., 2004) | Pakistanis | Non | 2004 |  |  |  |  |  |  |  |
| c.1214A>G | exon 7 | | p.His405Arg | (de Haar et al., 2005) | Not | Non | 2005 |  |  |  |  |  |  |  |
| c.1235A>G | exon 7 | | p.Tyr412Cys | (Hewitt et al., 2004) | Not | Non | 2004 |  |  |  |  |  |  |  |
| c.1268G>C | exon 7 | | p.Trp423Ser | (Noack et al., 2004) | German | Non | 2004 |  |  |  |  |  |  |  |
| c.1287G>C | exon 7 | | p.Trp429Cys | (Lefevre et al., 2001) | French | Non | 2001 |  |  |  |  |  |  |  |
| c.1337A>C | exon 7 | | p.Asp446Ala | (Wei et al., 2020) | Cambodian | Non | 2020 |  |  |  |  |  |  |  |
| c.1340A>G | exon 7 | | p.Glu447Gly | (Hewitt et al., 2004) | Not | Non | 2004 |  |  |  |  |  |  |  |
| c.1357A> G | exon 7 | | p.Ile453Val | (Nakano et al., 2001) | Not | Non | 2001 |  |  |  |  |  |  |  |
| c.1360A>G | exon 7 | | p.Glu447Gly | (P. S. Hart et al., 2000) | Vietnamese | Non | 2000 |  |  |  |  |  |  |  |
| **Variant type: Nonsense variant** | | | |  | | | | |  |  |  |  |  |  |
| c.72C>A | exon 1 | | p.Cys24* | (Lefevre et al., 2001) | Moroccan | Consanguineous | 2001 |  |  |  |  |  |  |  |
| c.90C >A | exon 1 | | p.Cys30* | (Nitta et al., 2005) | Thai | Non | 2005 |  |  |  |  |  |  |  |
| c.96T>G | exon 1 | | p.Tyr32* | (Lefevre et al., 2001) | French | Non | 2001 |  |  |  |  |  |  |  |
| c.145C>T | exon 1 | | p.Gln49* | (Selvaraju et al., 2003) | Indian | Non | 2003 |  |  |  |  |  |  |  |
| c.205C> T | exon 2 | | p.Gln69* | (Selvaraju et al., 2003) | Indian | Non | 2003 |  |  |  |  |  |  |  |
| c.322A>T | exon 3 | | p.Lys108* | (Bullon et al., 2014) | Italian | Non | 2014 |  |  |  |  |  |  |  |
| c.504C>G | exon 4 | | p.Tyr168* | (Bullon et al., 2014) | Italian | Non | 2014 |  |  |  |  |  |  |  |
| c.544C>T＃ | exon 4 | | p.Gln182* | (W. Wu et al., 2016) | Chinese | Non | 2016 |  |  |  |  |  |  |  |
| c.555G>A | exon 4 | | p.Trp185* | (P. S. Hart et al., 2002) | Brazilian | Consanguineous | 2002 |  |  |  |  |  |  |  |
| c.628C>T | exon 4 | | p.Arg210* | (Toomes et al., 1999) | Not | Non | 1999 |  |  |  |  |  |  |  |
| c.704G>A | exon 5 | | p.Trp235* | (P. S. Hart et al., 2000) | Iranian | Non | 2000 |  |  |  |  |  |  |  |
| c.711G>A | exon 5 | | p.Asp237* |  | Egyptian | Non | 2015 |  |  |  |  |  |  |  |
| c.748C>T＃ | exon 5 | | p.Arg250* | (P. S. Hart et al., 2000) | Turkish | Non | 2000 |  |  |  |  |  |  |  |
| c.754C>T＃ | exon 5 | | p.Gln252* | (Yuanjiao & Chen-Jun, 2016) | Chinese | Non | 2016 |  |  |  |  |  |  |  |
| c.856C>T | exon 6 | | p.Gln286* | (T. C. Hart et al., 1999) | Turkish | Consanguineous | 1999 |  |  |  |  |  |  |  |
| c.912C>A | exon 7 | | p.Tyr304* | (Selvaraju et al., 2003) | Indian | Non | 2003 |  |  |  |  |  |  |  |
| c.1035C>A＃ | exon 7 | | p.Tyr345* |  | Chinese |  | 2019 |  |  |  |  |  |  |  |
| c.1131T>G＃ | exon 7 | | p.Tyr377* | (Wen et al., 2012) | Chinese | Non | 2012 |  |  |  |  |  |  |  |
| c.1269G>A | exon 7 | | p.Trp423* | (Noack et al., 2008) | Sri Lanka | Consanguineous | 2008 |  |  |  |  |  |  |  |
| c.1286G>A | exon 7 | | p.Trp429* | (P. S. Hart et al., 2000) | Turkish | Non | 2000 |  |  |  |  |  |  |  |
| c.1287G>A | exon 7 | | p.Trp429* | (Decruyenaere et al., 1999) | Turkish | Consanguineous | 1999 |  |  |  |  |  |  |  |
| c.1339G>T | exon 7 | | p.Glu447* | (Moura et al., 2020) | Caucasian | Consanguineous | 2020 |  |  |  |  |  |  |  |
| **Variant type: Frameshift (Deletion variant)** | | | | | | | | |  |  | | |  |  |
| c.21delG | exon 1 | | p.Leu7fs | (Kurban et al., 2010) | Pakistani | Consanguineous | 2010 |  |  |  |  |  |  |  |
| c.103_105del | exon 1 | | p.Leu35fs | (Moghaddasian et al., 2014) | Iranian | Consanguineous | 2013 |  |  |  |  |  |  |  |
| c.112_115del＃ | exon 1 | | p.Thr38fs | (Yang et al., 2007) | Chinese | Non | 2007 |  |  |  |  |  |  |  |
| c.118delG＃ | exon 1 | | p.Val40fs | (X. Li et al., 2008) | Chinese | Non | 2008 |  |  |  |  |  |  |  |
| c.267_268del | exon 2 | | P.Asn89fs | (Pallos, Acevedo, Mestrinho, Cordeiro, & Hart, 2010) | Brazilian | Consanguineous | 2010 |  |  |  |  |  |  |  |
| c.436delT | exon 3 | | p.Ser146fs | (Noack et al., 2008) | German | Non | 2008 |  |  |  |  |  |  |  |
| c.566_572del | exon 4 | | p.Thr189fs | (Noack et al., 2008) | Russian | Non | 2008 |  |  |  |  |  |  |  |
| c.629_630del | exon 4 | | p.Arg210fs | (Wani, Devkar, Patole, & Shouche, 2006) | Indian | Non | 2006 |  |  |  |  |  |  |  |
| c.681_687del | exon 4 | | p.Thr227fs | (Farkas et al., 2013) | Hungarian | Non | 2013 |  |  |  |  |  |  |  |
| c.711_724del | exon 5 | | p.Trp237fs | (Lefevre et al., 2001) | Algerian | Consanguineous | 2001 |  |  |  |  |  |  |  |
| c.984_990del | exon 7 | | p.Cys331fs | (Lefevre et al., 2001) | French | Consanguineous | 2001 |  |  |  |  |  |  |  |
| c.1028_1029del | exon 7 | | p.Ser343fs | (T. C. Hart et al., 1999) | Turkish | Consanguineous | 1999 |  |  |  |  |  |  |  |
| c.1047delA | exon 7 | | p.Gly347fs | (T. C. Hart et al., 1999) | Turkish | Consanguineous | 1999 |  |  |  |  |  |  |  |
| c.1056delT | exon 7 | | p.Tyr352fs | (Lefevre et al., 2001) | French | Non | 2001 |  |  |  |  |  |  |  |
| c.1141delC | exon 7 | | p.Leu381fs | (Lefevre et al., 2001) | French | Non | 2001 |  |  |  |  |  |  |  |
| c.1211_1212del＃ | exon 7 | | p.Asn404fs | (Y. Wu et al., 2019) | Chinese | Non | 2019 |  |  |  |  |  |  |  |
| c.1213_1215del | exon 7 | | p.His405fs | (Wani et al., 2006) | Indian | Consanguineous | 2006 |  |  |  |  |  |  |  |
| **Variant type: Splice-site variant** | | | |  | | | | |  |  |  |  |  |  |
| c.318-1G>A |  | |  | (Hattab & Amin, 2005) | Jordanian | Consanguineous | 2005 |  |  |  |  |  |  |  |
| C.485-1G>A | intron 3 | |  | (Toomes et al., 1999) | Not | Non | 1999 |  |  |  |  |  |  |  |
| c.757-1G>A | intron 5 | |  | (Jouary et al., 2008) | Moroccan | Consanguineous | 2008 |  |  |  |  |  |  |  |
| c.757+1G>A＃ | intron 5 | |  | (Y. Wu et al., 2019) | Chinese | Non | 2019 |  |  |  |  |  |  |  |
| C.890-1G>T | intron 6 | |  | (Castori et al., 2009) | Italian | Non | 2009 |  |  |  |  |  |  |  |
| **Variant type: Small-insertion variant** | | | | | | | | |  |  | | |  |  |
| c.180_181insG | exon 2 | |  | (Hewitt et al., 2004) | Not | Non | 2004 |  |  |  |  |  |  |  |
| c.190_191insA＃ | exon 2 | |  | (Y. Wu et al., 2019) | Chinese | Non | 2019 |  |  |  |  |  |  |  |
| c.315dupT | exon 2 | |  | (Kobayashi, Sugiura, Takeichi, & Akiyama, 2013) | Japanese | Consanguineous | 2013 |  |  |  |  |  |  |  |
| c.366_367insGAC | exon 3 | |  | (Ghanei, Abbaszadegan, Forghanifard, Aarabi, & Arab, 2021) | Iranian | Non | 2021 |  |  |  |  |  |  |  |
| c.445_446insATGT | | exon 3 |  | (P. S. Hart et al., 2000) | Bengali | Non | 2000 |  |  |  |  |  |  |  |
| c.622_623insC | exon 4 | |  | (P. S. Hart et al., 2000) | Turkish | Non | 2000 |  |  |  |  |  |  |  |
| **Variant type: Gross deletion variant** | | | |  | | | | |  |  |  |  |  |  |
| c.199_222del＃ | exon 2 | |  | (P. S. Hart et al., 2000) | Chinese | Non | 2000 |  |  |  |  |  |  |  |
| g.88032292-88142997del＃ |  | |  | (W. Wu et al., 2016) | Chinese | Non | 2016 |  |  |  |  |  |  |  |
| g.88016961-88165581del |  | |  | (Schackert et al., 2014) | Not | Non | 2014 |  |  |  |  |  |  |  |
| Gene deletion exons 3–7 |  | |  | (Jouary et al., 2008) | Not | Non | 2008 |  |  |  |  |  |  |  |
| **Variant type: Regulatory variant** | | | |  | | | | |  |  |  |  |  |  |
| c.-55C>A | 5'-UTR | |  | (Kosem et al., 2012) | Slovenian | Non | 2012 |  |  |  |  |  |  |  |

Not: We don’t know which country the patients come from.

Non: The patient come from nonconsanguineous family or we don’t know the patients whether come from nonconsanguineous family

＃: These variants also had been reported in Chinese PLS patients

Reference

Allende, L. M., Garcia-Perez, M. A., Moreno, A., Corell, A., Carasol, M., Martinez-Canut, P., & Arnaiz-Villena, A. (2001). Cathepsin C gene: First compound heterozygous patient with Papillon-Lefevre syndrome and a novel symptomless mutation. Hum Mutat, 17(2), 152-153. doi:10.1002/1098-1004(200102)17:2<152::AID-HUMU10>3.0.CO;2-#

Allende, L. M., Moreno, A., & de Unamuno, P. (2003). A genetic study of cathepsin C gene in two families with Papillon-Lefevre syndrome. Molecular Genetics and Metabolism, 79(2), 146-148. doi:10.1016/s1096-7192(03)00070-2

Bullon, P., Morillo, J. M., Thakker, N., Veeramachaneni, R., Quiles, J. L., Ramirez-Tortosa, M. C., . . . Battino, M. (2014). Confirmation of oxidative stress and fatty acid disturbances in two further Papillon-Lefevre syndrome families with identification of a new mutation. J Eur Acad Dermatol Venereol, 28(8), 1049-1056. doi:10.1111/jdv.12265

Castori, M., Madonna, S., Giannetti, L., Floriddia, G., Milioto, M., Amato, S., & Castiglia, D. (2009). Novel CTSC mutations in a patient with Papillon-Lefevre syndrome with recurrent pyoderma and minimal oral and palmoplantar involvement. British Journal of Dermatology, 160(4), 881-883. doi:10.1111/j.1365-2133.2008.08878.x

Cury, V. F., Costa, J. E., Gomez, R. S., Boson, W. L., Loures, C. G., & De Marco, L. (2002). A Novel Mutation of the Cathepsin C Gene in Papillon-Lefevre Syndrome. J Periodontol, 73(3), 307-312. doi:10.1902/jop.2002.73.3.307

de Haar, S. F., Jansen, D. C., Schoenmaker, T., De Vree, H., Everts, V., & Beertsen, W. (2004). Loss-of-function mutations in cathepsin C in two families with Papillon-Lefevre syndrome are associated with deficiency of serine proteinases in PMNs. Hum Mutat, 23(5), 524. doi:10.1002/humu.9243

de Haar, S. F., Mir, M., Nguyen, M., Kazemi, B., Ramezani, G. H., Everts, V., & Beertsen, W. (2005). Gene symbol: CTSC. Disease: Papillon-Lefevre syndrome. Hum Genet, 116(6), 545. Retrieved from <https://www.ncbi.nlm.nih.gov/pubmed/15991336>

Decruyenaere, M., Evers-Kiebooms, G., Boogaerts, A., Cassiman, J. J., Cloostermans, T., Demyttenaere, K., . . . Fryns, J. P. (1999). Psychological functioning before predictive testing for Huntington's disease: the role of the parental disease, risk perception, and subjective proximity of the disease. J Med Genet, 36(12), 897-905. Retrieved from <https://www.ncbi.nlm.nih.gov/pubmed/10593997>

Farkas, K., Paschali, E., Papp, F., Valyi, P., Szell, M., Kemeny, L., . . . Csoma, Z. (2013). A novel seven-base deletion of the CTSC gene identified in a Hungarian family with Papillon-Lefevre syndrome. Arch Dermatol Res, 305(5), 453-455. doi:10.1007/s00403-013-1323-z

Ghanei, M., Abbaszadegan, M. R., Forghanifard, M. M., Aarabi, A., & Arab, H. (2021). A novel mutation in the cathepsin C (CTSC) gene in Iranian family with Papillon-Lefevre syndrome. Clin Exp Dent Res. doi:10.1002/cre2.387

Hart, P. S., Pallos, D., Zhang, Y., Sanchez, J., Kavamura, I., Brunoni, D., & Hart, T. C. (2002). Identification of a novel cathepsin C mutation (p.W185X) in a Brazilian kindred with Papillon-Lefevre syndrome. Molecular Genetics and Metabolism, 76(2), 145-147. doi:10.1016/s1096-7192(02)00031-8

Hart, P. S., Zhang, Y., Firatli, E., Uygur, C., Lotfazar, M., Michalec, M. D., . . . Hart, T. C. (2000). Identification of cathepsin C mutations in ethnically diverse papillon-Lefevre syndrome patients. J Med Genet, 37(12), 927-932. doi:10.1136/jmg.37.12.927

Hart, T. C., Hart, P. S., Bowden, D. W., Michalec, M. D., Callison, S. A., Walker, S. J., . . . Firatli, E. (1999). Mutations of the cathepsin C gene are responsible for Papillon-Lefevre syndrome. J Med Genet, 36(12), 881-887. Retrieved from <https://www.ncbi.nlm.nih.gov/pubmed/10593994>

Hattab, F. N., & Amin, W. M. (2005). Papillon-Lefevre syndrome with albinism: a review of the literature and report of 2 brothers. Oral Surg Oral Med Oral Pathol Oral Radiol Endod, 100(6), 709-716. doi:10.1016/j.tripleo.2004.08.030

Hayashi, M., Nakano, H., Sawamura, D., & Suzuki, T. (2020). Japanese case of Papillon-Lefevre syndrome with novel compound heterozygous mutations. J Dermatol, 47(8), e293-e295. doi:10.1111/1346-8138.15412

Hewitt, C., McCormick, D., Linden, G., Turk, D., Stern, I., Wallace, I., . . . Thakker, N. (2004). The role of cathepsin C in Papillon-Lefevre syndrome, prepubertal periodontitis, and aggressive periodontitis. Hum Mutat, 23(3), 222-228. doi:10.1002/humu.10314

Hu, T. T., Zou, X. Y., & Ye, F. (2019). [Gene mutational analyses of cathepsin C gene in a family with Papillon-Lefevre syndrome]. Hua Xi Kou Qiang Yi Xue Za Zhi, 37(1), 31-36. doi:10.7518/hxkq.2019.01.006

Jouary, T., Goizet, C., Coupry, I., Redonnet-Vernhet, I., Levade, T., Burgelin, I., . . . Arveiler, B. (2008). Detection of an intragenic deletion expands the spectrum of CTSC mutations in Papillon-Lefevre syndrome. J Invest Dermatol, 128(2), 322-325. doi:10.1038/sj.jid.5700987

Kobayashi, T., Sugiura, K., Takeichi, T., & Akiyama, M. (2013). The novel CTSC homozygous nonsense mutation p.Lys106X in a patient with Papillon-Lefevre syndrome with all permanent teeth remaining at over 40 years of age. British Journal of Dermatology, 169(4), 948-950. doi:10.1111/bjd.12429

Kosem, R., Debeljak, M., Repic Lampret, B., Kansky, A., Battelino, T., & Trebusak Podkrajsek, K. (2012). Cathepsin C gene 5'-untranslated region mutation in papillon-lefevre syndrome. Dermatology, 225(3), 193-203. doi:10.1159/000342509

Kurban, M., Cheng, T., Wajid, M., Kiuru, M., Shimomura, Y., & Christiano, A. M. (2010). A novel mutation in the cathepsin C gene in a Pakistani family with Papillon-Lefevre syndrome. J Eur Acad Dermatol Venereol, 24(8), 967-969. doi:10.1111/j.1468-3083.2010.03575.x

Lefevre, C., Blanchet-Bardon, C., Jobard, F., Bouadjar, B., Stalder, J. F., Cure, S., . . . Fischer, J. (2001). Novel point mutations, deletions, and polymorphisms in the cathepsin C gene in nine families from Europe and North Africa with Papillon-Lefevre syndrome. J Invest Dermatol, 117(6), 1657-1661. doi:10.1046/j.0022-202x.2001.01595.x

Li, X., Zhang, X., Zhang, J., & Chen, Y. (2008). [Mutational analysis of the cathepsin C gene in a family of Han nationality with Papillon-Lefevre syndrome]. Zhonghua Yi Xue Yi Chuan Xue Za Zhi, 25(5), 502-505. Retrieved from <https://www.ncbi.nlm.nih.gov/pubmed/18841559>

Li, Z., Liu, J., Fang, S., Zhu, H., Zhang, X., Cai, J., . . . Xu, Y. (2014). Novel compound heterozygous mutations in CTSC gene cause Papillon-Lefevre syndrome with high serum immunoglobulin E. J Dermatol Sci, 76(3), 258-260. doi:10.1016/j.jdermsci.2014.09.009

Machado, R. A., Cuadra-Zelaya, F. J. M., Martelli-Junior, H., Miranda, R. T., Casarin, R. C. V., Correa, M. G., . . . Coletta, R. D. (2019). Clinical and molecular analysis in Papillon-Lefevre syndrome. American Journal of Medical Genetics Part A, 179(10), 2124-2131. doi:10.1002/ajmg.a.61285

Meenu, S., Pradeep, B., Ramalingam, S., Sairam, T., Rai, R., & Sankaran, R. (2020). Papillon-Lefevre syndrome (PLS) with novel compound heterozygous mutation in the exclusion and Peptidase C1A domains of Cathepsin C gene. Molecular Biology Reports, 47(7), 5681-5687. doi:10.1007/s11033-020-05622-0

Moghaddasian, M., Arab, H., Dadkhah, E., Boostani, H., Babak, A. R., & Abbaszadegan, M. R. (2014). Protein modeling of cathepsin C mutations found in Papillon-Lefevre syndrome. Gene, 538(1), 182-187. doi:10.1016/j.gene.2013.11.079

Molitor, A., Prud'homme, T., Miao, Z., Conrad, S., Bloch-Zupan, A., Pichot, A., . . . Carapito, R. (2019). Exome sequencing identifies a novel missense variant in CTSC causing nonsyndromic aggressive periodontitis. Journal of Human Genetics, 64(7), 689-694. doi:10.1038/s10038-019-0615-3

Moura, A. L., Regateiro, F. S., Peres Resende, E., Coimbra Silva, H., Goncalo, M., Todo Bom, A., & Faria, E. (2020). A New Terminal Nonsense Mutation of the Cathepsin C Gene in a Patient With Atypical Papillon-Lefevre Syndrome. J Investig Allergol Clin Immunol, 30(2), 151-153. doi:10.18176/jiaci.0467

Nakano, A., Nomura, K., Nakano, H., Ono, Y., LaForgia, S., Pulkkinen, L., . . . Uitto, J. (2001). Papillon-Lefevre syndrome: mutations and polymorphisms in the cathepsin C gene. J Invest Dermatol, 116(2), 339-343. doi:10.1046/j.1523-1747.2001.01244.x

Nitta, H., Wara-Aswapati, N., Lertsirivorakul, J., Nakamura, T., Yamamoto, M., Izumi, Y., . . . Ishikawa, I. (2005). A novel mutation of the cathepsin C gene in a thai family with Papillon-Lefevre syndrome. J Periodontol, 76(3), 492-496. doi:10.1902/jop.2005.76.3.492

Noack, B., Gorgens, H., Hoffmann, T., Fanghanel, J., Kocher, T., Eickholz, P., & Schackert, H. K. (2004). Novel mutations in the cathepsin C gene in patients with pre-pubertal aggressive periodontitis and Papillon-Lefevre syndrome. Journal of Dental Research, 83(5), 368-370. doi:10.1177/154405910408300503

Noack, B., Gorgens, H., Schacher, B., Puklo, M., Eickholz, P., Hoffmann, T., & Schackert, H. K. (2008). Functional Cathepsin C mutations cause different Papillon-Lefevre syndrome phenotypes. J Clin Periodontol, 35(4), 311-316. doi:10.1111/j.1600-051X.2008.01201.x

Ochiai, T., Nakano, H., Rokunohe, D., Akasaka, E., Toyomaki, Y., Mitsuhashi, Y., & Sawamura, D. (2009). Novel p.M1T and recurrent p.G301S mutations in cathepsin C in a Japanese patient with Papillon-Lefevre syndrome: implications for understanding the genotype/phenotype relationship. J Dermatol Sci, 53(1), 73-75. doi:10.1016/j.jdermsci.2008.07.006

Pallos, D., Acevedo, A. C., Mestrinho, H. D., Cordeiro, I., & Hart, T. C. (2010). Novel cathepsin C mutation in a Brazilian family with Papillon-Lefevre syndrome: case report and mutation update. J Dent Child (Chic), 77(1), 36-41. Retrieved from <https://www.ncbi.nlm.nih.gov/pubmed/20359428>

Romero-Quintana, J. G., Frias-Castro, L. O., Arambula-Meraz, E., Aguilar-Medina, M., Duenas-Arias, J. E., Melchor-Soto, J. D., . . . Ramos-Payan, R. (2013). Identification of novel mutation in cathepsin C gene causing Papillon-Lefevre Syndrome in Mexican patients. BMC Med Genet, 14, 7. doi:10.1186/1471-2350-14-7

Schackert, H. K., Agha-Hosseini, F., Gorgens, H., Jatzwauk, M., von Kannen, S., Noack, B., . . . Mehdipour, P. (2014). Complete homozygous deletion of CTSC in an Iranian family with Papillon-Lefevre syndrome. Int J Dermatol, 53(7), 885-887. doi:10.1111/j.1365-4632.2012.05769.x

Selvaraju, V., Markandaya, M., Prasad, P. V., Sathyan, P., Sethuraman, G., Srivastava, S. C., . . . Kumar, A. (2003). Mutation analysis of the cathepsin C gene in Indian families with Papillon-Lefevre syndrome. BMC Med Genet, 4, 5. doi:10.1186/1471-2350-4-5

Sorensen, O. E., Clemmensen, S. N., Dahl, S. L., Ostergaard, O., Heegaard, N. H., Glenthoj, A., . . . Borregaard, N. (2014). Papillon-Lefevre syndrome patient reveals species-dependent requirements for neutrophil defenses. Journal of Clinical Investigation, 124(10), 4539-4548. doi:10.1172/JCI76009

Tekin, B., Yucelten, D., Beleggia, F., Sarig, O., & Sprecher, E. (2016). Papillon-Lefevre syndrome: report of six patients and identification of a novel mutation. Int J Dermatol, 55(8), 898-902. doi:10.1111/ijd.13297

Toomes, C., James, J., Wood, A. J., Wu, C. L., McCormick, D., Lench, N., . . . Thakker, N. S. (1999). Loss-of-function mutations in the cathepsin C gene result in periodontal disease and palmoplantar keratosis. Nat Genet, 23(4), 421-424. doi:10.1038/70525

Wang, X., Liu, Y., Liu, Y., Dong, G., Kenney, E. B., Liu, Q., . . . Wang, Q. (2015). Long-term change of disease behavior in Papillon-Lefevre syndrome: seven years follow-up. Eur J Med Genet, 58(3), 184-187. doi:10.1016/j.ejmg.2014.12.003

Wani, A. A., Devkar, N., Patole, M. S., & Shouche, Y. S. (2006). Description of two new cathepsin C gene mutations in patients with Papillon-Lefevre syndrome. J Periodontol, 77(2), 233-237. doi:10.1902/jop.2006.050124

Wei, H., Wee, L. W. Y., Born, B., Seang, S., Koh, M. J. A., Yee, R., . . . Tan, E. C. (2020). Palmoplantar keratoderma, oral involvement, and homozygous CTSC mutation in two brothers from Cambodia. American Journal of Medical Genetics Part A, 182(2), 296-302. doi:10.1002/ajmg.a.61447

Wen, X., Wang, X., & Duan, X. (2012). High immunoglobulin E in a Chinese Papillon-Lefevre syndrome patient with novel compound mutations of cathepsin C. J Dermatol, 39(7), 664-665. doi:10.1111/j.1346-8138.2011.01474.x

Wu, W., Chen, B., Chen, X., Chen, L., Yi, L., Wang, Y., . . . Sun, W. (2016). A novel large deletion combined with a nonsense mutation in a Chinese child with Papillon-Lefevre syndrome. J Periodontal Res, 51(3), 376-380. doi:10.1111/jre.12317

Wu, Y., Zhao, L., Xu, C., & Wu, Y. (2019). CTSC compound heterozygous mutations in two Chinese patients with Papillon-Lefevre syndrome. Oral Dis, 25(5), 1394-1402. doi:10.1111/odi.13095

Yang, Y., Bai, X., Liu, H., Li, L., Cao, C., & Ge, L. (2007). Novel mutations of cathepsin C gene in two Chinese patients with Papillon-Lefevre syndrome. Journal of Dental Research, 86(8), 735-738. doi:10.1177/154405910708600809

Yang, Y., Bai, X. W., Liu, H. S., Cao, C. F., & Ge, L. H. (2006). [Novel mutations of cathepsin C gene in two Chinese patients with Papillon-Lefevre syndrome]. Zhonghua Kou Qiang Yi Xue Za Zhi, 41(10), 602-605. Retrieved from <https://www.ncbi.nlm.nih.gov/pubmed/17129448>

Yuanjiao, C., & Chen-Jun, L. (2016). [Gene mutational analyses of the cathepsin C gene in families with Papillon-Lefevre syndrome]. Hua Xi Kou Qiang Yi Xue Za Zhi, 34(4), 346-349. doi:10.7518/hxkq.2016.04.005

Zhang, Y., Hart, P. S., Moretti, A. J., Bouwsma, O. J., Fisher, E. M., Dudlicek, L., . . . Hart, T. C. (2002). Biochemical and mutational analyses of the cathepsin c gene (CTSC) in three North American families with Papillon Lefevre syndrome. Hum Mutat, 20(1), 75. doi:10.1002/humu.9040

Zhang, Y., Lundgren, T., Renvert, S., Tatakis, D. N., Firatli, E., Uygur, C., . . . Hart, T. C. (2001). Evidence of a founder effect for four cathepsin C gene mutations in Papillon-Lefevre syndrome patients. J Med Genet, 38(2), 96-101. doi:10.1136/jmg.38.2.96
